# Video based educational intervention in waiting area to improve awareness about health screening among patients visiting family medicine clinics

**DOI:** 10.1186/s12913-024-11143-4

**Published:** 2024-07-16

**Authors:** Rabeeya Saeed, Farah Ahmed, Syed Hasan Danish, Mohammad Talha, Maha Usmani, Noureen Durrani, Noman Ali

**Affiliations:** 1 Family Medicine, Liaquat National Hospital, Karachi, Pakistan; 2 Community Health Sciences, Ziauddin University, Karachi, Pakistan; 3 Liaquat National Hospital and Medical College, Karachi, Pakistan; 4 Publication Department, Liaquat National Hospital, Karachi, Pakistan

**Keywords:** Health screening, Educational intervention, Family medicine, Diabetes mellitus, Hypertension, Cancer

## Abstract

**Background:**

Multiple educational modalities have been utilized including leaflet, face-to-face counseling and watching videos in waiting areas for engaging patients. Considering the two challenges of waiting time frustration and lack of health screening awareness, Family Physicians’ waiting area are an ideal place to bridge this gap. The objective of this study is to evaluate the effectiveness of video-based health education intervention in improving knowledge about health screening among patients and their families sitting in waiting area of Family Medicine clinics.

**Methods:**

It was a pre and post quasi-experimental study that was conducted in family medicine clinics located at main campus and Outreach centers of a tertiary care hospital. A total of 300 participants were approached during the six month period. The intervention consisted of an educational video on health screening. The content of the video was taken from the recommended preventive care guidelines from CDC and USPSTF. The pre-and post-intervention knowledge of the participants was assessed through a semi-structured coded questionnaire by an interviewer who was trained in data collection. Data was analyzed using SPSS version 26. Pre and post intervention knowledge adequacy was determined using MacNemar’s Chi-square test.

**Results:**

Total 300 participants voluntarily participated into the study. Median age of the participants was 28 (IQR = 23.25–36.75) years. Majority of participants were males (56%). Following the intervention, there was significant increase in the proportion of participants (51.3% versus 68%) who had understanding of health screening check-up (*p* < 0.001). Following the study intervention, there was significant increase in proportion of participants who had adequate knowledge related to diabetes (*p* = 0.045), hypertension (*p* < 0.001), cholesterol (*p* < 0.001), cervical cancer (*p* < 0.001), colon cancer (*p* < 0.001) and hepatitis B & C (*p* < 0.001). No significant improvement in breast cancer related knowledge was observed (*p* = 0.074). Highest post-intervention increase in knowledge from baseline was observed for hypertension (13.3% versus 63.3%) followed by colon cancer (24.3% versus 59.3%), cholesterol (67 versus 96.7%), hepatitis b & C (56.7% versus 77.3%), diabetes (29.7% versus 48%), cervical cancer (1.7% versus 19%), and breast cancer (7.7% versus 18.3%).

**Conclusion:**

This study highlighted a pivotal role of an educational video intervention in clinic waiting area to improve awareness regarding health screening among patients and their families. Further interventional community based or multicenter studies are warranted to assess the long-term impact of these educational videos on knowledge and utilization of health screening among adult population.

**Supplementary Information:**

The online version contains supplementary material available at 10.1186/s12913-024-11143-4.

## Background

Health screening is an effective strategy towards early diagnosis of diseases in asymptomatic individuals with a goal to prevent complication and death from disease [1, 2]. As reported in literature, almost 85% of women’s death due to cervical cancer in lower middle-income countries was attributed to lower screening rates [3]. Lack of knowledge and awareness remains a major barrier which ultimately limits the utilization of screening services among general population [4].

Contrary to the system prevailing in developed countries where 90% of population has screening coverage by health care system funded by the government; developing countries like Pakistan lacks any data reporting the frequency of population undergoing health screening for diseases like diabetes, hypertension and various cancers. This highlights the importance and role of primary care health providers to make their clients aware of routine health screening.

Multiple educational modalities including written leaflet, face-to-face counseling and watching videos have been utilized to impart health education when a patient visit health care facility. However Educational videos proved to more effective than written materials especially for people with low health literacy at enhancing knowledge and modifying health behaviors. A meta-analysis has proved effectiveness of videos in breast self-examination, prostate cancer screening, sunscreen adherence, self-care in patients with heart failure, HIV testing, treatment adherence, and female condom use [5].

A video intervention was successful in enhancing knowledge regarding stroke symptoms and satisfaction with education in admitted stroke patients [6]. Numerous community based educational programs also utilized videos to impart education on inhaler technique, COPD and asthma [7]. A multimedia-based educational program not only increased the awareness regarding cervical cancer screening from a proportion of women with good knowledge from 2 to 70.5% but also enhanced the utilization of screening services (from 4.3 to 8.3%) [8].

In our health care system where no formal screening services are engraved in health care system opportunistic advice regarding health screening remains a real challenge in a busy clinic. The purpose of this study is to assess the feasibility of implementing video based educational intervention in doctor’s clinic waiting area evaluate patient’s baseline knowledge on health screening of non-communicable and infectious diseases and assess the impact of video based education on patient’s knowledge regarding health screening. The result of this study will be helpful in designing randomized controlled trial in future to determine effectiveness of video based intervention on health screening which may ultimately affect utilization of screening services by patients.

## Methods

### Study design, setting inclusion and exclusion criteria

It was a pre and post Quasi experimental study that was conducted in Family Medicine clinics located at main campus and Outreach centers of a tertiary care hospital. A total of 320 participants were approached during the six month period. A total of 300 gave consent to participate in the study who were enrolled through non-probability consecutive sampling. Patients who were very sick, in severe pain, vitally unstable, required Emergency or admission referral, were unable to understand Urdu and those who refused to participate in the study were excluded from the study.

### Educational intervention

An 8-minute educational video intervention was developed on health screening to be shown to the participants on TV screen installed in waiting area. The content was prepared by a faculty of Family Medicine using recommended preventive care guidelines from CDC and USPSTF [9, 10]. The video script was written in Urdu (local language) at a 7th grade reading level in order to facilitate wide range of literacy level. Background audio, simple animations and pictorial display of concepts were used to enhance practical understanding of participants. The concepts addressed in this video included health screening tests and their significance in preventive health care; Information about screening recommendations for following diseases.

- Non- Communicable diseases: hypertension, diabetes, dyslipidemia.
- Cancers: breast, cervical and colon cancers.
- Infections including Hepatitis B and C.

For each of these diseases, risk factors, available screening tests, appropriate age and frequency of screening were displayed. The video was revised based on the feedback of people from diverse field including patients, their family members, nursing staff, non-clinical administrative staff who assessed it for sound effects and understandability of concepts. The final version was verified by two experts from Family Medicine and Public Health.

### Study instrument

The pre-and post-intervention knowledge of the participants was assessed through a semi structured coded questionnaire by an interviewer who was trained in data collection. The questionnaire was designed by principal investigator after thorough literature search and the content was validated by other two experts of family medicine who were not part of this study. The questionnaire consisted of three parts including 34 items in all. The first part (Questions 1–9) gathered information about socio demographics including age, gender, socioeconomic status, and education level and comorbid conditions. Second part (Question 10–29) assessed knowledge about general concept of health screening tests. Knowledge about each disease screening was assessed through 2–3 questions. To minimize the risk of bias each question had at least 4 options to choose from. Each correct answer was assigned a score of 1 and wrong items were recorded as zero. Total score for knowledge related to diabetes, hypertension, high cholesterol, breast cancer, colon cancer and Hepatitis B & C was 5, 3, 8, 4, 4 and 5 respectively. The third part (Question 30–34) assessed utilization of health screening services by patients and barriers related to non-utilization of these services (on Likert scale). Pilot study was performed on 30 patients for testing questionnaire before collecting final sample. Cronbach’s alpha was computed for all knowledge components and for third part of questionnaire. All values of the Cronbach’s alpha were ≥ 0.70 and then initial draft was finalized as in its original form. According to Bloom’s criteria, knowledge was considered as adequate for each component when participants scored at least 80% of total score in that component [11]. The study questionnaire is attached as supplementary file.

### Data collection procedure

**Fig. 1:**
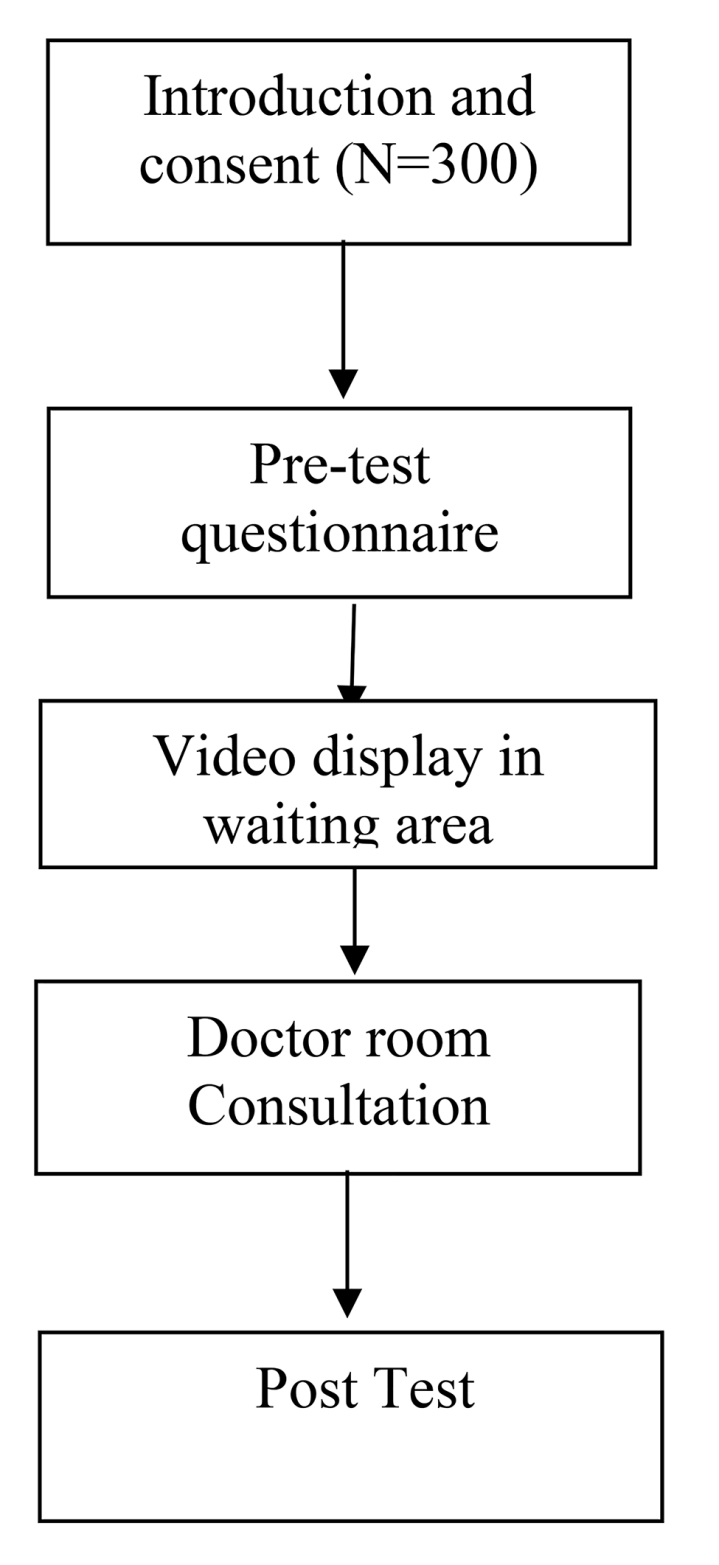
Flow chart for the intervention study

Patients and their families were approached in the assessment area prior to their doctor’s visit to confirm eligibility for their participation and their willingness to participate in the study. Those who gave written consent to participate were interviewed to complete a pre-test questionnaire and after that they were shown a video on health screening at least once in the waiting area. After doctor’s consultation they filled the post- test questionnaire prior to leaving from clinic (Fig. 1).

### Sample size calculation

Pilot study was performed on 30 subjects to estimate sample size calculation. NCSS PASS version 11 was used to perform sample size calculation with option of tests for two correlated proportions (McNemar’s Test). Sample size was separately estimated at 95% confidence interval and 80% power for diabetes, hypertension, cholesterol, breast cancer, cervical cancer, colon cancer and hepatitis part. Proportions that we obtained in pilot study presented in Table 1. The highest calculated sample size was 246. However, for better results we enrolled 300 patients.

**Table 1:** Frequency of correct responses before and after intervention

Knowledge components	P1	P2	Discordant pairs proportion	Sample size
Diabetes	0.117	0.300	0.417	96
Hypertension	0.520	0.020	0.540	15
Cholesterol	0.313	0.017	0.330	28
Breast cancer	0.150	0.043	0.193	130
Cervical cancer	0.177	0.300	0.477	246
Colon cancer	0.367	0.017	0.384	23
Hepatitis B & C	0.240	0.033	0.273	48

### Data analysis

Data was entered into IBM SPSS version 20 for statistical analysis. Frequencies and percentages were computed for categorical variables. Median and inter-quartile range was reported for age after testing normal distribution with Shaprio-Wilk test. Pre and post intervention knowledge adequacy was determined using MacNemar’s Chi-square test. A two tailed p-value < 0.05 was taken as statistically significant.

## Results

Total 300 participants voluntarily participated into the study with written consent. Median age of study participants was 28(23.25–36.75) years. Majority of participants were male (*n* = 168, 56%) Most of the study subjects completed secondary school or above (*n* = 105, 35%), were either graduate (*n* = 161, 53.7.7%). Few of the participants had religious education (*n* = 10, 3.3%), 21(7%) were primary pass and 3(1%) were illiterate. About half of the study participants were patients (*n* = 149, 49.7%) and the rest were attendants (*n* = 151, 50.3%).

Prior to intervention of video-based learning, nearly half of the participants had awareness of health screening check-up (*n* = 154, 51.3%) and following the intervention there was significant increase in the proportion of participants (*n* = 204, 68%) who had understanding of health screening check-up (*p* < 0.001) (Table 2).

There was significant increase in knowledge of the study participants for specific knowledge questions for all of diseases including non-communicable diseases (Table 3), cancer screening and risk factors (Table 4), infectious diseases screening and risk factors (Table 5).

**Table 2:** Frequency of correct responses before and after intervention

Knowledge Question	Pre-intervention *n*(%)	Post-intervention *n*(%)	*p*-value
What do you understand by health screening checkup?	154(51.3)	204(68)	< 0.001

**Table 3:** Screening of Non communicable disease

Adequate knowledge of Diabetes Screening	Pre-intervention *n*(%)	Post-intervention *n*(%)	*p*-value
Age for screening	83(27.7)	190(63.3)	< 0.001
Family history of Diabetes as risk factor	63(21)	293(97.7)	< 0.001
Family history of Asthma as risk factor	205(68.3)	247(82.3)	< 0.001
Obesity as risk factor	206(68.7)	281(93.7)	< 0.001
Depression as risk factor	136(45.3)	164(54.7)	0.001
Knowledge of hypertension			
Blood pressure threshold for identifying hypertension	166(55.3)	267(89)	< 0.001
Usually there are no symptoms of blood pressure	14(4.7)	96(32)	< 0.001
Frequency of blood pressure monitoring for healthy adults	52(17.3)	183(61)	< 0.001
Knowledge of Dyslipidemia Screening and risk factors			
Age for screening	177(59)	268(89.3)	< 0.001
Tobacco as risk factor	162(54)	262(87.3)	< 0.001
Being underweight as risk factor	163(54.3)	226(75.3)	< 0.001
Family history of depression as risk factor	64(21.3)	130(43.3)	< 0.001
Family history of high cholesterol as risk factor	254(84.7)	291(97)	< 0.001
Asthma as risk factor	126(42)	214(71.3)	< 0.001
High blood pressure as risk factor	247(82.3)	287(95.7)	< 0.001
Depression as risk factor	68(22.7)	127(42.3)	< 0.001
Diabetes as risk factor	248(82.7)	290(96.7)	< 0.001
Knowledge related to complications of diabetes and hypertension			
Diabetes and hypertension can silently damage the eyes, kidneys, cause heart problems and stroke	214(71.3)	284(94.7)	< 0.001

**Table 4:** Knowledge of Cancer Screening and Risk factors

Knowledge domains for different malignancies	Pre-intervention *n*(%)	Post-intervention *n*(%)	*p*-value
Breast Cancer			
Age for mammogram screening	83(27.7)	74(24.7)	0.380
Family history as risk factor	168(56)	218(72.7)	< 0.001
Mammography as recommended method for screening	126(42)	229(76.3)	< 0.001
Cervical cancer			
Age for cervical cancer screening	81(27)	126(42)	< 0.001
Test for cervical cancer screening	71(23.7)	202(67.3)	< 0.001
Frequency of cervical cancer screening	30(10)	117(39)	< 0.001
Colon cancer			
Family history as risk factor	135(45)	205(68.3)	< 0.001
Test for colon cancer screening	133(44.3)	258(86)	< 0.001

**Table 5:** Knowledge for Infectious Disease Screening and Risk factors

Hepatitis B and C related knowledge	Pre-intervention *n*(%)	Post-intervention *n*(%)	*p*-value
Transmission through sexual relationship	224(74.7)	253(84.3)	< 0.001
Transmission through pregnancy	226(75.3)	253(84.3)	< 0.001
Transmission through affected person	184(61.3)	229(76.3)	< 0.001
Transmission through eating with affected person	155(51.7)	227(75.7)	< 0.001
Test for Hepatitis B & C screening?	211(70.3)	269(89.7)	< 0.001

**Fig. 2:**
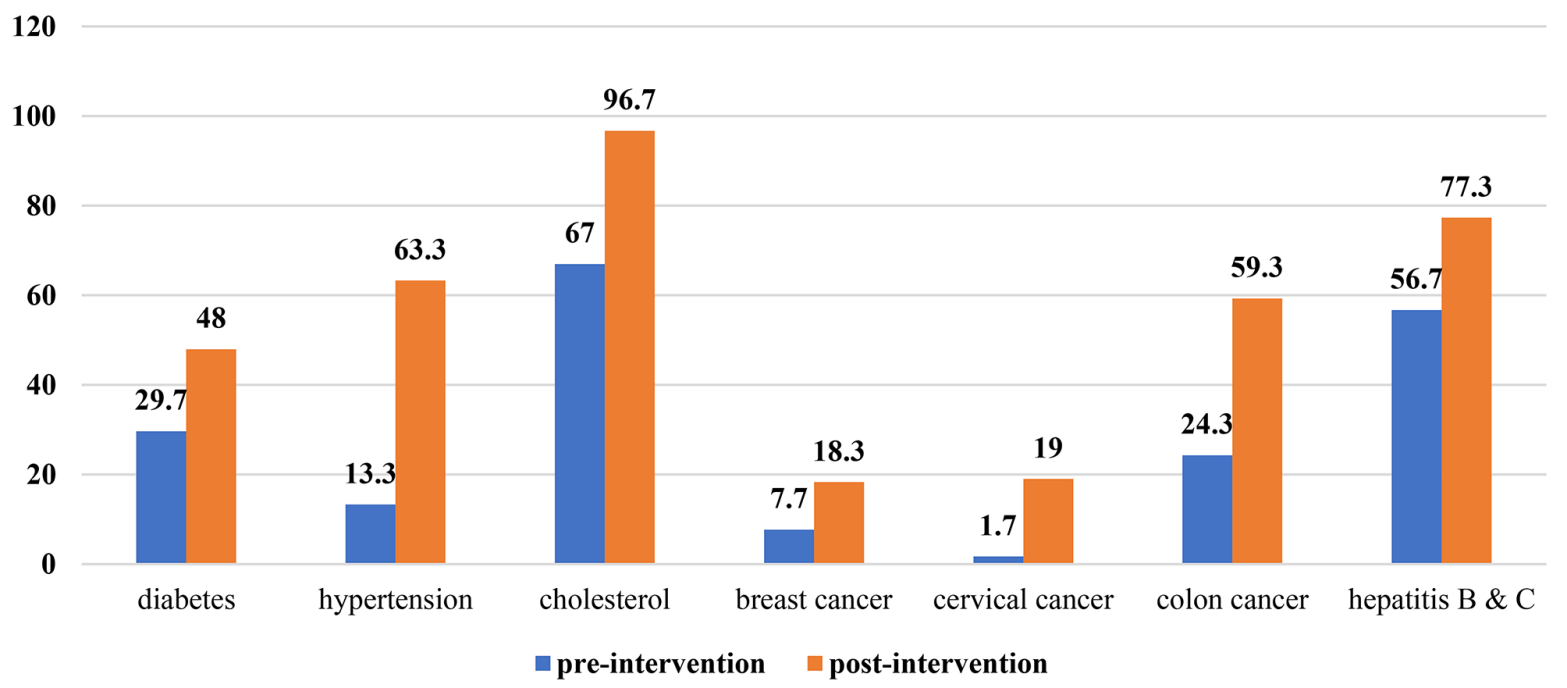
Frequency of adequate knowledge before and after intervention

Figure 2 is depicting the frequency of adequate knowledge on different diseases before and after intervention. Prior to the intervention, among all disease, the frequency of adequate knowledge was high for cholesterol (*n* = 201, 67%) followed by hepatitis B & C (*n* = 170, 56.7%), diabetes (*n* = 89, 29.3%), colon cancer (*n* = 73, 24.3%), hypertension (*n* = 40, 13.3%), breast cancer (*n* = 23, 7.7%) and cervical cancer (*n* = 5, 1.7%). Following the study intervention, there was significant increase in proportion of participants who had adequate knowledge related to diabetes (*p* = 0.045), hypertension (*p* < 0.001), cholesterol (*p* < 0.001), cervical cancer (*p* < 0.001), colon cancer (*p* < 0.001) and hepatitis B & C (*p* < 0.001). No significant improvement in breast cancer related knowledge was observed (*p* = 0.074). Highest post-intervention increase in knowledge from baseline was observed for hypertension (13.3% versus 63.3%) followed by colon cancer (24.3% versus 59.3%), cholesterol (67 versus 96.7%), hepatitis b & C (56.7% versus 77.3%), diabetes (29.7% versus 48%), cervical cancer (1.7% versus 19%), and breast cancer (7.7% versus 18.3%).

**Fig. 3:**
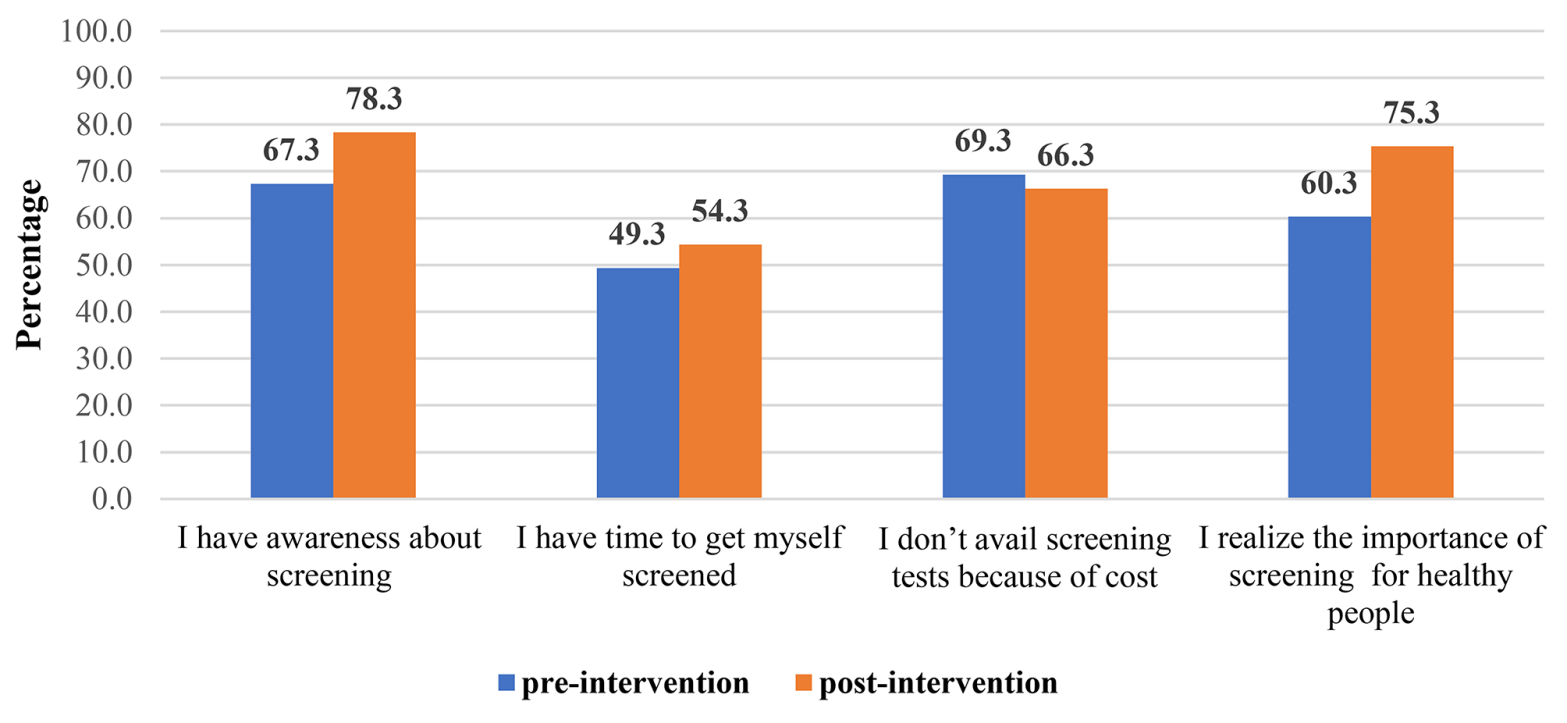
Attitude regarding utilization of health screening services pre and post intervention

Figure 3 is displaying the participants’ attitude towards health screening check-up before and after intervention. Prior to watching the knowledge-based video, 202 (67.3%) respondents considered themselves unaware of health screening, and after intervention, 235 (78.3%) participants reported that they had awareness (*p* < 0.001). Before intervention about half of the participants (*n* = 148, 49.3%) had no priority for health screening due to busy schedule and their attitude significantly improved after intervention (*n* = 163, 54.3%) (*p* = 0.024). 208(69.3%) reported that the reason of not availing screening test is because of cost of the procedures, and after intervention this proportion further decreased (*n* = 199, 69.3%) but it was not significant (*p* = 0.150). In perception of 181(60.3%) participants, they did not think health screening is essential; following the intervention, there was significant improvement in their way of thinking (*p* < 0.001) and 226 (75.3%) considered it as important.

## Discussion

Video intervention has been reported in the literature as an effective strategy to increase knowledge and awareness regarding isolated disease screening like cervical cancer, breast cancer etc. Our study adopted a novel approach by implementing a video intervention encompassing holistic approach to adult health screening. The intervention was targeted to impart education regarding the diseases for which screening tests are recommended for general adult population as per international guidelines.

Majority of the participants were formally educated however, their baseline knowledge regarding concept of health screening and risk factors of non-communicable (diabetes, Hypertension, dyslipidemia, cancers) and infectious diseases was very low. This is understandable due to the traditional illness-based approach prevailing among common people, seeking health care only when symptoms occur. Another reason could be the absence of established preventive health care programs by the government at primary care level.

Before the video intervention there was a decreased awareness of risk factors and screening of hypertension (13.3%) and diabetes (297%). These results are comparable with the study conducted among African Americans which showed an increase in knowledge of diabetic screening after electronic health intervention [12]. In 2021, a study was done in India upon a population of 64,427 people aged 45 years and above to assess the awareness, treatment, and control (ATC) of hypertension which showed a low awareness (54.4%) among the population leading to barriers in optimal treatment of hypertension in the general population [13]. This is nevertheless a higher percentage than found in our study and is alarming that such a small proportion of individuals were aware of hypertension screening in our study. This could be attributed to the silent nature of disease, there being no definite symptoms marking the severity of blood pressure control.

Our study showed a good baseline knowledge regarding dyslipidemia (67%) which improved significantly after the intervention (96.7%). This is a satisfying statistics; in contrast, our literature search yielded a less than 50% awareness regarding their cholesterol screening [14].

Pre and post video intervention in our study regarding knowledge of hypertension, diabetes and dyslipidemia suggested that there was substantial knowledge gain. Similar type of studies showed an increase in knowledge and attitude in people with hypertension after using video presentation [15]. One study also showed an enhancement upon the impact of hypertension control after using multimedia as a teaching tool for general public [16]. These results imply that educational intervention using multimedia as an educational medium can favorably affect the awareness and acceptability of hypertension screening among the masses and may have a key role in improving the knowledge scores regarding both hypertension screening and later on, its control.

Our study participants had a good baseline awareness regarding hepatitis B and C screening (56.7%) which improved after the intervention (77.3%). Though there is paucity of research evaluating the impact of video education on Hepatitis screening; however two recent studies showed similar results [17, 18].

1.7% of the population were aware of cervical cancer screening before the video intervention, which improved to 19.3% post-intervention in the present study. A study including 600 participants in Ghana depicted much higher percentages before (84.2%) and after (100%) a video intervention [19]. Other studies also depict a significant improvement in cervical cancer screening awareness among the women after an educational intervention [20–23].

The low baseline awareness with a lesser increase in awareness post intervention could be attributable to majority of male participants in our study which may have lesser interest in women related cancers.

Our study depicted that 24.3% of the participants were aware of colorectal screening methods before the intervention, while post-intervention, it improved to 59.3%.These figures are comparable to another study done in Khyber pakhtunkhuan in 2021 where 32% had no knowledge regarding colorectal cancer screening [24].

Breast cancer is a growing menace internationally with 76.7% incidence rate in Pakistan [25]. The results of a study published in 2022 upon 774 university students from different universities of all 4 provinces of Pakistan show 44.4% awareness of the correct age of mammography as 40 years. 29.8% demonstrated an understanding of family history as a recognized risk factor for the development of breast cancer, while the study did not assess the correct mode of breast cancer screening [26]. 42% of the participants demonstrated an understanding of mammography as a reliable screening tool for breast cancer before the study intervention, which improved to 76.3% post-intervention. However, increase in overall knowledge of breast cancer after intervention was lowest among all other diseases. The reason of lower knowledge may be that majority were males in this study which might be not concerned over female related issues.

Similar results were found in a systemic review demonstrating increase awareness regarding different regarding prevention and screening of different cancers including breast cancer [27].

We found that implementing the educational video in the outpatient clinic waiting area was feasible. Over 95% of the consented patients completed the intervention. The main challenge that was encountered was distraction of noise and talking of other people sitting in the waiting area which may compromise the attention span of the participant. Use of headphone devices to counteract this challenge can be a suitable option for future. In addition, we aimed to allocate the same time for video watching to each participant; however this could not be materialized due to variability of waiting time and consultation of patients at variable pace.

Our study population included both patients and their accompanied family members. The mean age of our study population was 28 with a range from 23 to 36 years old. More than 80% of study population had education above secondary level and graduate. Therefore, we believe that the video effectiveness cannot be determined for people with low education level as their health literacy may be inadequate to understand the concepts presented in this video.

We found that this simple video based intervention had influenced the perception regarding health screening. More than half of the participants (60%) who perceived health screening as non- essential pre- intervention became sensitized to its importance and there was a significant increase in the number of participants (75%) who now considered it as integral component of health maintenance. However cost was a barrier to utilization of health screening which remained unchanged after video intervention.

These findings are in concordance with other studies that investigated the impact of video-intervention upon improving the attitudes and acceptability regarding health screening [28].

Our study has some limitations. Being single arm and non-randomized there was no comparison group or comparing intervention like written material to assess the most effective approach towards health education. Moreover, these was no control on patients’ waiting time and number of time they had watched the video but it was assured that they watched the video at least one time. Another noticeable point is that in this study, we interviewed patients in a private designated area instead of self-administering the questionnaire which may impact the results. Particularly, in Pakistani setting, patients visiting the clinics are more focused to consult their doctors and self-administering of questionnaire could cause lack of their attention because of filling out long questionnaires. That’s why it is better to interview them in our settings. The long term impact of this educational intervention was not determined. It’s worthwhile to explore long term impact of this intervention by evaluating the knowledge after 3–6 months. Moreover, we did not control for the variability in other sources of education that participants may already have owing to their variable background and health literacy level which may confound the results. In addition, our questionnaire has not undergone testing for validity. In addition the video impact has to be assessed for population differences in gender, ethnicity, and age and education level.

A randomized trial will clearly be needed to evaluate the efficacy of the video as a tool to improve knowledge regarding health screening. Limitations and findings of this study will be taken into consideration while designing our forthcoming randomized trial. Moreover, given the available evidence that repetition facilitates learning, the video will be shown to participants on multiple occasions with the opportunity to ask questions for long term knowledge retention.

## Conclusion

This study highlighted a pivotal role of an educational video intervention in clinic waiting area to improve awareness regarding health screening among patients and their families. However, the study may have resulted in over estimation of knowledge score since it was carried in a single hospital setting among patients and their families whose health seeking behaviors and health literacy may be different from general population. Further community-based or multicenter randomized controlled trials are warranted to assess the long term impact of these educational videos on knowledge and utilization of health screening among adult population.

### Electronic supplementary material

Below is the link to the electronic supplementary material.

Supplementary Material 1

## Data Availability

The data collected and analyzed for this study is presented in the article.

## Abbreviations

aOR: Adjusted odds ratio
ATC: Awareness, treatment, and control
CDC: Center for disease control
COPD: Chronic obstructive pulmonary disease
CVD: Cardiovascular disease
DASH: Dietary approach to stop hypertension
USPSTF: United States Preventive Services Task Force
