# Supplementary material for: Video based educational intervention in waiting area to improve awareness about health screening among patients visiting family medicine clinics
Source: BMC Health Serv Res. 2024 Jul 16;24:818. doi: 10.1186/s12913-024-11143-4 (PMC11253394; doi:10.1186/s12913-024-11143-4)
Supplement: Supplementary file 1 — Supplementary Material 1 [file 12913_2024_11143_MOESM1_ESM.docx]

|  | **Code:** | Date: | | | | | | | | | | | |  |
| --- | --- | --- | --- | --- | --- | --- | --- | --- | --- | --- | --- | --- | --- | --- |
|  | **Demographic details** |  | | | | | | | | | | | |  |
| 1. | Name(optional) |  | | | | | | | | | | | |  |
| 2. | Record no/centre |  | | | | | | | | | | | |  |
| 3. | Age |  | | | | | | | | | | | |  |
| 4. | Gender | 1. Male 2. Female | | | | | | | | | | | |  |
| 5. | Education | 1. Madrasa 2. Primary 3. Secondary 4. Graduate 5. Masters 6. No education | | | | | | | | | | | |  |
| 6. | Household income/month |  | | | | | | | | | | | |  |
| 7. | Employment status | 1. House wife 2. Office work 3. Business 4. Labour 5. Doctor 6. Engineer 7. Unemployed 8. Others | | | | | | | | | | | |  |
| 8 | Reason for visit | 1. Patient 2. Accompanying patient | | | | | | | | | | | |  |
| 9 | Co morbid illness | 1. Diabetes 2. High blood pressure 3. High cholesterol 4. Heart disease 5. Hepatitis B 6. Hepatitis C 7. Cancer 8. Other 9. None | | | | | | | | | | | |  |
|  | **Health screening** |  | | | | | | | | | | | | **Score** |
| 10. | What do you understand by health screening checkup? | 1. Tests that look for disease before you have symptoms 2. Tests that diagnose the disease after symptoms appear 3. Tests that show result on a computer screen | | | | | | | | | | | | 1 |
|  | **Diabetes** |  | | | | | | | | | | | |  |
| 11. | At what age a healthy person should be tested for diabetes even if there are no symptoms?  (Mark only one correct answer) | 1. 30-35 year 2. 40-45 year 3. 50-55 year 4. Should only get tested if there are symptoms | | | | | | | | | | | | 1 |
| 12. | In which conditions, you should be tested for Diabetes at an earlier age than recommended.  (Mark all options as Yes, No or don’t know) | Family history of Diabetes | | Yes | | No | | Don’t know | | | | | | 4 |
|  |  | Family history of Asthma | | Yes | | No | | Don’t know | | | | | |  |
|  |  | If you are overweight | | Yes | | No | | Don’t know | | | | | |  |
|  |  | Suffering from Depression | | Yes | | No | | Don’t know | | | | | |  |
|  | **Hypertension/High blood pressure** |  | | | | | | | | | | | |  |
| 13. | Which of the following reading is cut off level for diagnosis of high blood pressure?  (Mark only one correct answer) | 1. 120/80 2. 140/90 3. 130/80 4. 150/90 | | | | | | | | | | | | 1 |
| 14. | Which of the following is the most common presentation of high blood pressure?  (Mark only one correct answer) | 1. Vomiting 2. Headache 3. No symptoms 4. Abdominal pain 5. Don’t know | | | | | | | | | | | | 1 |
| 15. | How often a normal person (with no high blood pressure) should get his blood pressure routinely checked?  (Mark only one correct answer) | 1. Daily 2. Weekly 3. Monthly 4. Yearly | | | | | | | | | | | | 1 |
|  | **High cholesterol** |  | | | | | | | | | | | |  |
| 16. | Following people should check their blood cholesterol on regular basis | Those who use tobacco | | | | | Yes | | | No | | Don’t know | | 8 |
|  |  | Those who are underweight | | | | | Yes | | | No | | Don’t know | |  |
|  |  | Family history of depression | | | | | Yes | | | No | | Don’t know | |  |
|  |  | Family history of high cholesterol | | | | | Yes | | | No | | Don’t know | |  |
|  |  | Asthma | | | | | Yes | | | No | | Don’t know | |  |
|  |  | High blood pressure | | | | | Yes | | | No | | Don’t know | |  |
|  |  | Depression | | | | | Yes | | | No | | Don’t know | |  |
|  |  | Diabetes | | | | | Yes | | | No | | Don’t know | |  |
| 17. | At what age a normal person without any disease should start checking his blood cholesterol?  (Mark only one correct answer) | 1. 35 2. 45 3. 65 4. Don’t know | | | | | | | | | | | | 1 |
|  | **Complications of Diabetes and Hypertension** | | | | | | | | | | | | |  |
| 18. | Which of the following diseases can silently damage the eyes, kidneys, cause heart problems and stroke? | 1. Diabetes 2. Asthma 3. High blood pressure 4. Don’t know | | | | | | | | | | | | 2 |
|  | **Breast Cancer** | | | | | | | | | | | | |  |
| 19. | If a woman has family history of Breast Cancer (mother, sister, aunt), what happens to her chance of getting breast cancer?  (Mark only one answer) | 1. Increase 2. Decrease 3. No effect 4. Don’t know | | | | | | | | | | | | 1 |
| 20. | Which of the following are recommended methods for early detection of breast cancer?  (You can mark more than one option) | 1. Bone scan 2. Mammography 3. X ray chest 4. Breast self-examination | | | | | | | | | | | | 2 |
| 21. | At what age does a healthy woman should start regular Mammogram for screening of Breast cancer?  (Mark only one answer) | 1. 30 2. 40 3. 50 4. 60 | | | | | | | | | | | | 1 |
|  | **Cervical Cancer** | | | | | | | | | | | | |  |
| 22. | Have you heard of cervical cancer? | 1. Yes 2. No | | | | | | | | | | | | 1 |
| 23. | Which test can screen cervical cancer at an early stage? | 1. Pap/LBC test 2. Ultrasound 3. Colonoscopy 4. Don’t know | | | | | | | | | | | | 1 |
| 24. | At what age a sexually active woman should get routine testing for cervical cancer screening?  (Mark only one answer) | 1. 21 and above 2. 41 and above 3. Don’t know 4. 61 and above | | | | | | | | | | | | 1 |
| 25. | How frequent should this testing be done?  (Mark only one answer) | 1. Every year 2. Every 3 year 3. Every 6 year 4. Don’t know | | | | | | | | | | | | 1 |
|  | **Colon Cancer** | | | | | | | | | | | | |  |
| 26. | If a man has family history of colon cancer then what happ ens to his risk of getting colon cancer? | 1. Increase 2. Decrease 3. No change 4. Don’t know | | | | | | | | | | | | 1 |
| 27. | What are the recommended methods for colon cancer screening?  (You can mark more than one) | 1. Colonoscopy 2. CT Scan Abdomen 3. Stool for occult blood 4. Mammography | | | | | | | | | | | | 2 |
|  | **Hepatitis B and C** |  | | | | | | | | | | | |  |
| 28. | Which of the following are the routes of transmission for Hepatitis B and C? | From sexual relationship | | | Yes | | No | | | | Don’t know | | | 4 |
|  |  | From mother to child during delivery | | | Yes | | No | | | | Don’t know | | |  |
|  |  | Touching the affected person | | | Yes | | No | | | | Don’t know | | |  |
|  |  | Eating with the affected person | | | Yes | | No | | | | Don’t know | | |  |
| 29. | Which test is used to screen for Hepatitis B or C? | 1. Urine test 2. Blood test 3. Ultrasound liver 4. Don’t know | | | | | | | | | | | | 1 |
|  | **Utilization of Health screening tests** |  | | | | | | | | | | | |  |
| 33. | Have you ever screened/tested yourself for following diseases | Diabetes | | | Yes | | No | | | | Already diagnosed | | |  |
|  |  | High blood pressure | | | Yes | | No | | | | Already diagnosed | | |  |
|  |  | High cholesterol | | | Yes | | No | | | | Already  Diagnosed | | |  |
|  |  | Hepatitis B | | | Yes | | No | | | | Already diagnosed | | |  |
|  |  | Hepatitis C | | | Yes | | No | | | | Already diagnosed | | |  |
|  |  | Colon Cancer | | | Yes | | No | | | | Already diagnosed | | |  |
|  |  | Cervical Cancer (women only) | | | Yes | | No | | | | Already diagnosed | | |  |
|  |  | Breast Cancer  (women only) | | | Yes | | No | | | | Already diagnosed | | |  |
|  | If answer is **No** to any of the above-mentioned disease, Choose the reasons for not getting tested from below | | | | | | | | | | | | | |
| 31 | I don’t have awareness about screening | Strongly agree | Agree | | | | | | Disagree | | | | Strongly disagree | |
| 32 | I don’t have time to get myself screened | Strongly agree | Agree | | | | | | Disagree | | | | Strongly disagree | |
| 33 | I don’t avail screening tests because of cost | Strongly agree | Agree | | | | | | Disagree | | | | Strongly disagree | |
| 34 | I don’t realize the importance of screening for healthy people | Strongly agree | Agree | | | | | | Disagree | | | | Strongly disagree | |
